# Identification and diversity of multiresistant *Corynebacterium striatum *clinical isolates by MALDI-TOF mass spectrometry and by a multigene sequencing approach

**DOI:** 10.1186/1471-2180-12-52

**Published:** 2012-04-04

**Authors:** Margarita Gomila, Feliu Renom, Maria del Carmen Gallegos, Margarita Garau, Dolores Guerrero, Joan B Soriano, Jorge Lalucat

**Affiliations:** 1 Unidad de Investigación-Microbiología, Fundación Hospital Son Llàtzer, Ctra. Manacor, km. 4, 07198 Palma de Mallorca, Illes Balears, Spain; 2 Respiratory Unit, Hospital Joan March, Ctra. Sóller, km. 12, 07110 Bunyola, Illes Balears, Spain; 3 Microbiology Service, Fundación Hospital Son Llàtzer, Ctra. Manacor, km. 4, 07198 Palma de Mallorca, Illes Balears, Spain; 4 Program of Epidemiology and Clinical Research, Fundació Caubet-CIMERA Illes Balears, International Centre for Advanced Respiratory Medicine, Ctra. Sóller, km. 12, 07110 Bunyola, Illes Balears, Spain; 5 Microbiologia, Departament de Biologia, Universitat de les Illes Balears, and Institut Mediterrani d'Estudis Avançats (CSIC-UIB), Ctra. Valldemossa, km. 7.5, 07122 Palma de Mallorca, Illes Balears, Spain

## Abstract

**Background:**

The genus *Corynebacterium *is composed of Gram-positive bacteria that are widely distributed throughout the environment; these bacteria are also part of the normal microbiota of human skin and mucous membranes. Multiple studies have shown that species of this genus, including *C. striatum*, become pathogenic to humans under special conditions. Our aim was to determine the characteristics of clinical multiresistant strains of *C. striatum *that were isolated in our geographical region, to determine their diversity, and to compare them with the type strain and with related species. We studied fifty-two strains of *C. striatum *isolated from different hospitals from Mallorca, Spain, mainly from the Hospital Joan March in Bunyola, Mallorca. Most of the strains were isolated from sputum cultures of respiratory samples from patients with chronic obstructive pulmonary disease. To gain further insight into the genetic diversity of the strains, we analysed several housekeeping genes and other genes associated with antibiotic resistance. Strains were also characterised phenotypically by their antibiotic resistance profiles and by MALDI-TOF mass spectrometry analysis.

**Results:**

The ITS1 region, *gyrA *and *rpoB *were chosen as the appropriate genes in the *C. striatum *genome to study the genetic diversity of *C. striatum *species and to discriminate between strains. After analysing these three genes, four sequence types (ST2, ST4, ST1 and ST11) were found to be the most abundant. Splits tree analysis of the strains demonstrated that these clinical isolates did not share any alleles with the type strain of the species. Recombination was detected within all of the *C. striatum *isolates, and different clonal populations were detected within the samples.

**Conclusions:**

Our results demonstrate that the isolates were best identified using gene-based molecular methods; using these methods, the isolated strains were determined to be different from the type strain of *C. striatum*. The ITS1 region and the *gyrA *and *rpoB *genes were selected because of their variability and were the most useful tools for discriminating between strains. The phenotype and antibiotype characteristics of the strains did not seem suitable for typing purposes. MALDI-TOF mass spectrometry can be a useful method for identifying and discriminating between *C. striatum *strains.

## Background

The genus *Corynebacterium *includes pathogens, non-pathogenic environmental bacteria, and saprophytic species. The most widely known pathogenic species is *C. diphtheriae. C. diphtheriae*, endemic in many countries, represents a global health problem because of the outbreaks it has caused in recent decades, as documented by the WHO. Characterisation of the strains is needed to obtain a better understanding and microbiological and epidemiological control [1]. In addition to *C. diphtheriae*, other potentially pathogenic species of the genus are *C. amycolatum, C. jeikeium, C. macginleyi *and *C. urealyticum *[2-4]. *C. xerosis *has also been described as an unusual pathogen [5]. Outbreaks of nosocomial infections have been reported for *C. pseudodiphtheriticum *[6-8] and, remarkably, *C. striatum *[9-12].

*C. striatum *is widely disseminated in the environment and constitutes part of the normal microbiota of the skin and mucous membranes. However, it is potentially pathogenic in specific circumstances, including in infections of patients with lasting chronic diseases, frequent and prolonged hospitalisations, exposure to antibiotics against Gram-negative bacteria (which facilitates the selection of Gram positives), the use of invasive procedures and the presence of organic obstructive pathologies [11,12]. Any circumstance wherein there is increased longevity of disease or chronic disease increases the risk of infection and results in infections occurring more frequently.

Although the significance and prevalence of *C. striatum *as a causative agent of disease are not well understood, this organism has been responsible for a variety of different infections [11,13]. Most *C. striatum *infections reported to date have been found in respiratory samples, with the vast majority of the strains being multiresistant to antibiotics. Leonard *et al. *and Bradenburg *et al. *studied the presence of *C. striatum *in intensive care units, postulating the existence of person-to-person transmission [9,10]. Otsuka *et al. *[11] described the frequent isolation of *C. striatum *in long-stay advanced diseases that were subjected to repeated antibiotic courses. In 2007, Renom *et al. *[12] described the first nosocomial outbreak of this bacterium in patients with chronic obstructive pulmonary diseases (COPD). All of the strains identified in this outbreak were antibiotic multiresistant.

To understand the source of an outbreak, it is very important to have reliable identification and typing methods for the responsible bacteria. Several studies have tried to accomplish this objective [10,11], but none of them employ a methodology for the identification and typing of bacterial strains. The main aim of our study is to determine the parameters for characterisation of clinical multiresistant strains of *C. striatum *from sputum cultures isolated from the respiratory samples of COPD patients in our geographical region; we aim to determine the diversity of the strains and to compare the isolates with the *C. striatum *type strain and with related species.

All strains were characterised phenotypically by RapID CB^® ^Plus strips (Remel Laboratories, Lenexa, KS), by their antibiotic susceptibility profile and also by genomic profiling (ERIC-PCR, Enterobacterial Repetitive Intergenic Consensus-PCR). These experimental methods provided limited resolution. To gain further insight into the diversity of the *C. striatum *strains, a multilocus sequence typing (MLST) scheme was developed to identify significant intraspecies genetic diversity. MLST, proposed in 1998 by Maiden *et al. *[14], has shown that nucleotide variation within several core metabolic genes provides portable, reproducible and high-resolution data appropriate for evolutionary and epidemiological investigations. The strains were also analysed using matrix-assisted laser desorption ionisation time-of-flight (MALDI-TOF) mass spectrometry. MALDI-TOF has been reported by several studies as a powerful tool with accurate and reproducible results for rapid identification of clinical isolates in the microbiology laboratory. This method is simple, rapid, easy to perform, inexpensive and may ultimately replace routine phenotypic assays [15,16].

## Methods

### *C. striatum *culture collection

A total of 52 strains of *C. striatum *(collected between May 2006 and June 2009) were studied from three hospitals located in Mallorca, Spain. All of these strains were analysed and compared with the type strain of *C. striatum *ATCC 6940^T ^and the type strain of *C. amycolatum *CCUG 35685^T^, the closest-related species; the isolated strains were also compared with two strains from the culture collection of the Göteborg University (CCUG) that were characterised in a first approach as *C. striatum *strains (one from a clinical origin and the other environmental). All *Corynebacterium *strains were isolated and cultured on Columbia agar with 5% sheep blood (bioMérieux). Prior to cultivation, all samples were Gram-stained to determine the samples that could be discarded; strains that were not representative of the lower respiratory tract and the ones contaminated with microbiota from the upper respiratory tract, according to the Murray and Washington criteria, were not used [17]. The cultivation and incubation of the plates were performed under routine laboratory conditions. All of the strains are shown as Additional file 1: Table S1.

### Phenotypical and antibiotic susceptibility characterisations

The 56 strains were analysed phenotypically by RapID CB Plus^® ^strips, and their antibiogram profiles were established by E-test assay (AB Biodisk, Solna, Sweden) on Mueller-Hinton agar plates supplemented with 5% of blood (bioMérieux, Marcy d'Etoile, France), according to CLSI recommendations [18].

### DNA extraction: PCR amplification and DNA sequencing

Bacterial genomic DNA for PCR amplifications was obtained as previously described [19]. All *C. striatum *strains were compared by the ERIC-PCR technique, using the primers ERIC1R (5'-ATGTAAGCTCCTGGGGATTCAC-3') and ERIC2 (5'-AAGTAAGTGACTGGGGTGAGCG-3') [20]. The housekeeping genes, 16S rDNA, ITS1 (internal transcribed spacer 1), *gyrB, hsp65, rpoB *and *sodA*, were amplified and sequenced for the 56 strains. Two genes codifying for antibiotic resistance, *aphA *and *ermX*, were also amplified and sequenced for these strains. Three other primer sets codifying for antibiotic resistances (*cmx, repB *and *tetA*) were also tested but did not produce an amplicon. The list of primers is indicated as Additional file 2: Table S2 [21-25]. PCR amplification and sequence reaction was performed as previously described [19].

### Allele diversity, nucleotide diversity and statistical analysis

Allele and nucleotide diversities were calculated from the gene sequences with the DnaSP package, version 3.51 [26]. For identification purposes, distinct allele sequences were assigned arbitrary allele numbers for each locus. For each isolate, the combination of alleles obtained at each locus defined its allelic profile. Each allelic profile constitutes a sequence type (ST), and isolates with identical profiles belonged to the same ST. Clustering of STs was performed with the Sequence Type Analysis and Recombinational Tests (START) program [27]. The matrix of pair-wise distances between the allelic profiles was converted to NEXUS files, and the split decomposition was analysed with the SplitsTree software program, vs. 4 [28]. Splits tree allowed researchers to visualise clustering within the population and to detect recombination between STs. The nucleotide sequences determined in this study for the different alleles of each locus have been deposited in the EMBL database under the accession numbers HE586270 to HE586309.

### Analysis by MALDI-TOF mass spectrometry

Matrix-assisted linear desorption/ionisation-time-of-flight mass spectrometry (MALDI-TOF MS) analyses for all strains were performed at Anagnostec, GmbH, Germany [29], as described Scotta *et al. *[30].

## Results

### Phenotypic characterisation and antibiotic susceptibility tests of the isolates

All colonies were pale yellow in colour, nonhemolytic, catalase positive and oxidase negative. The strains were identified by the RapID CB Plus^® ^strips as *C. striatum *(51 strains with a confidence level between 85.54% - 99.97%), *C. pseudodiphtheriticum *(2 strains with a 100% of confidence level), or *C. amycolatum *(1 strain with a confidence level of 51.26%) [Additional file 3: Table S3]. All isolates were susceptible to vancomycin and resistant to cefotaxime and ciprofloxacin, whereas susceptibilities to other antibiotics tested were heterogeneous (Additional file 4: Table S4). The type strain of *C. amycolatum *was susceptible to all the antibiotics tested. The *C. striatum *type strain was susceptible to all of the antibiotics except cefotaxime. The two isolates that were analysed from the CCUG were sensitive to antibiotics.

### ERIC-PCR

Groupings based on the most similar fingerprint type of ERIC-PCR generated seven different profiles (represented by strains 2, 11, 15, 19, 31, 60 and 70) for *C. striatum *strains. The profile of the type strain of *C. striatum *was different from those of the clinical isolates; differences between the isolates were also observed (see Additional file 5: Figure S1).

### Multilocus sequence typing

Seven genes were determined for most of the strains studied. The 16S rRNA gene was excluded from the exhaustive analysis because of the high conservation between all of the strains studied; it was only used as a control to check the authenticity of the strains. Clinical isolates 16 and 17, characterised by phenotypical methods as *C. pseudodiphtheriticum*, were affiliated with the *C. striatum *species as determined by molecular methods. The *ermX, aphA *and *sodA *genes were also excluded from the analysis because of the high conservation between all strains.

The ITS1, *gyrA *and *rpoB *genes were used to discriminate between strains, although the genes differed at few nucleotide changes within the sequences. The sequence analysis of ITS1 demonstrated the presence of more than one *rrn *operon in most of the strains, which was not appreciable in the agarose gel as a double band but was detectable in the sequence electropherogram. The presence of more than one operon was checked by cloning of four PCR products (data not shown). Analysis of the *gyrA *and *rpoB *genes revealed that the variability between different *Corynebacterium *species occurred throughout the gene, while the variability in the clinical *C. striatum *isolates was confined to certain areas near the beginning of the gene.

Distinct allele sequences were assigned arbitrary allele numbers for each locus (Table 1). Calculated allele and nucleotide diversities are shown in Table 2. The number of polymorphic sites and the haplotype and nucleotide diversity were not calculated for the ITS1 region because, in most cases, more than one operon was detected. 16S rDNA, *ermX, aphA, sodA *and *hsp65 *were not appropriate genes for studying the genetic diversity of the strains, although these genes could be used to differentiate between *Corynebacterium *species. *gyrA *and *rpoB *were appropriate genes to study genetic diversity, with 116 and 39 polymorphic sites, respectively. In the ITS1 region, the most abundant alleles were 4 (23.2%), 6 (19.6%), 7 (12.5%), 3 (10.7%), and alleles 1 and 2 (7.1%). Each one of the other alleles for ITS1, representing 19.6% of the population, is represented by a single strain. For the *gyrA *gene, two alleles (number 2 and 3) were predominant (90%). For the *rpoB *gene, allele 2 is the most abundant and is found in 39 strains (69.6%). Considering these three genes, four STs were the most abundant: ST2, ST4, ST1 and ST11, occurring in 11, 10, 6 and 6 strains, respectively.

**Table 1:** STs at the eight loci examined in the *C. striatum *and *C. amycolatum *strains studied

Strain	16Sr DNA	ITS1	*gyrA*	*rpoB*	*hsp65*	*sodA*	*ermX*	*aphA*	ST*
**2**	1	3	2	2	1	1	1	1	1
**7**	1	3	2	2	1	1	1	1	1
**9**	1	6	2	2	1	-	1	1	2
**11**	1	1	3	2	1	1	1	1	3
**12**	1	1	3	2	1	-	1	1	3
**14**	1	6	2	2	1	1	1	1	2
**15**	1	4	3	2	1	-	1	1	4
**16**	1	4	3	2	1	1	1	1	4
**17**	1	2	2	2	1	1	1	1	5
**18**	1	4	3	2	1	-	1	1	4
**19**	1	1	3	2	1	-	1	1	3
**21**	1	6	2	2	1	1	1	1	2
**23**	1	6	2	2	1	1	1	1	2
**24**	1	2	2	2	1	1	1	-	5
**25**	1	3	2	2	1	1	1	1	1
**26**	1	6	2	2	1	1	1	1	2
**28**	1	7	3	2	1	1	1	1	6
**29**	1	5	2	2	1	1	1	1	7
**30**	1	4	3	4	1	1	1	1	8
**31**	1	6	2	2	1	1	1	1	2
**35**	1	6	2	2	1	-	1	1	2
**36**	1	6	2	2	1	1	1	1	2
**41**	1	6	2	2	1	1	1	1	2
**42**	1	6	2	2	1	-	1	1	2
**43**	1	3	2	2	1	-	1	-	1
**44**	1	4	3	2	1	-	1	1	4
**46**	1	3	2	2	1	1	1	1	1
**47**	1	4	3	2	1	1	1	1	4
**48**	1	2	2	2	1	1	1	-	5
**50**	1	4	3	2	1	1	1	1	4
**51**	1	10	4	2	1	-	1	-	9
**53**	1	2	2	2	1	1	1	1	5
**54**	1	3	2	2	1	1	1	-	1
**55**	1	6	2	2	1	1	1	1	2
**56**	1	1	3	2	1	-	1	1	3
**57**	1	4	3	2	1	1	1	1	4
**58**	1	4	3	2	1	1	1	1	4
**59**	1	4	3	2	1	-	1	1	4
**60**	1	4	3	2	1	1	1	1	4
**61**	1	12	3	3	1	1	1	-	10
**62**	1	7	3	3	1	-	1	1	11
**63**	-	7	3	3	1	-	1	1	11
**64**	1	7	3	3	1	1	1	-	11
**65**	1	7	3	3	1	-	1	-	11
**66**	1	4	3	4	1	1	1	1	8
**67**	1	7	3	3	1	1	1	-	11
**68**	1	7	3	3	1	-	1	-	11
**69**	1	9	5	4	2	1	1	-	12
**70**	2	13	7	6	3	2	1	1	13
**71**	1	11	4	2	1	1	1	1	14
**73**	1	4	3	4	1	1	1	-	8
**74**	1	8	3	4	1	-	1	2	15
***C. striatum *ATCC 6940^T^**	1	17	1	1	1	-	1	-	16
***C. amycolatum *CCUG 35685^T^**	5	14	3	7	4	-	-	-	17
**CCUG 39137**	3	15	3	6	3	-	-	2	18
**CCUG 44705**	4	16	6	5	1	-	-	-	19

* ST number was obtained taking into account only three of the eight loci analyzed (ITS1 region, *gyrA*, and *rpoB *genes)

**Table 2:** Genetic diversity of the selected loci among the *Corynebacterium *strains analysed

No. of strains	Locus	Fragment length (bp)^£^	No. of alleles	Haplotype (gene) diversity ± SD	No. of polymorphic sites	Avg number of nucleotide differences	Nucleotide diversity ± SD
56 (48)	16S rDNA	872	5 (1)	0.722 ± 0.159	71 (0)	31.972	0.037 ± 0.007
56 (49)	ITS1^¥^	360^¥^	17 (10)	-	-	-	-
56 (49)	*gyrA*	200	7 (3)	0.604 ± 0.040 (0.527 ± 0.001)	39 (3)	2.452 (1.073)	0.012 ± 0.006 (0.005 ± 0.000)
56 (49)	*rpoB*	380	7 (3)	0.498 ± 0.076 (0.379 ± 0.079)	116 (4)	11.551 (0.912)	0.0314 ± 0.012 (0.002 ± 0.000)
56 (49)	*hsp65*	287	4 (1)	0.138 ± 0.062 (0 ± 0)	62 (0)	6.199 (0)	0.022 ± 0.011 (0 ± 0)
35 (32)	*sodA*	295	2 (1)	0.057 ± 0.053 (0 ± 0)	64 (0)	3.657 (0)	0.012 ± 0.011 (0 ± 0)
53 (49)	*ermX*	367	1 (1)	0 ± 0 (0 ± 0)	0 (0)	0 (0)	0 ± 0 (0 ± 0)
41 (38)	*aphA*	333	2 (2)	0.095 ± 0.061 (0.053 ± 0.049)	1 (1)	0.095 (0.053)	0.0003 ± 0.0002 (0 ± 0)

The values corresponding only to clinical strains of *C. striatum *are given within brackets

^£ ^The number indicated the length of the sequenced fragment used for the polymorphic analysis

^¥ ^The number of polymorphic sites, and the haplotype and nucleotide diversity was not calculated for the ITS1 region because in most cases more than one operon was detected. The number of alleles was established directly from the alignment

**Figure 1:**
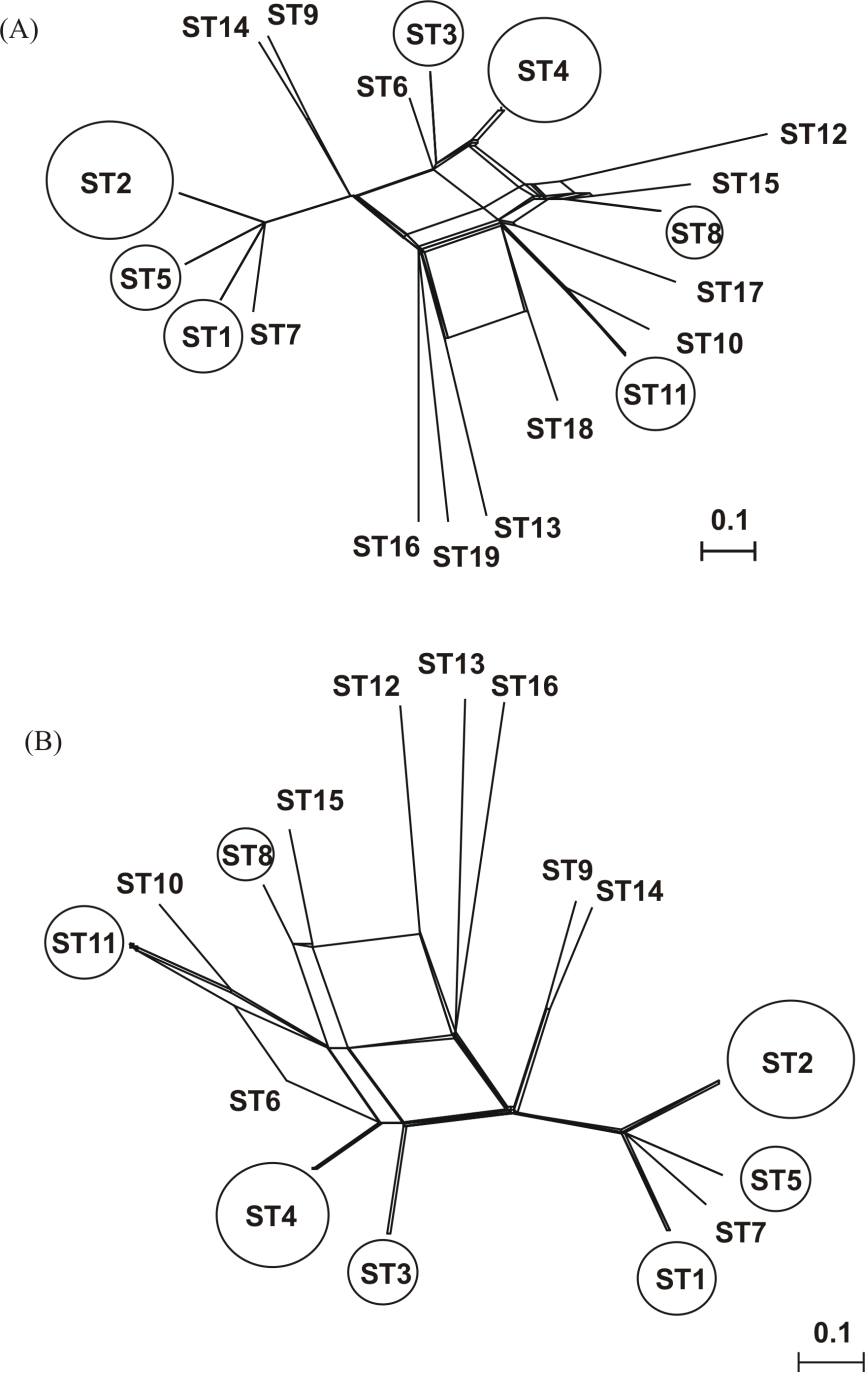
**Splits tree showing the distribution of all of sequence types obtained**. Splits tree was based on the ITS1, *gyrB *and *rpoB *genes allelic profile, for all analysed strains (panel **A**), and only for the *C. striatum *strains (panel **B**). The circles indicated the sequence types represented by more than one strain. The size of the circle is proportional to the number of strains included in each sequence type.

To determine the possible lateral gene transfers occurring in the population of *Corynebacterium *strains studied and taking into account the ITS1, *gyrB *and *rpoB *gene allelic profiles, a splits tree was constructed for all analysed strains (Figure 1, panel A) and only with the *C. striatum *strains (Figure 1, panel B). Some strains have two or three identical genes. Type strains of *C. striatum *and *C. amycolatum *did not share any allele, and recombination was detected between all of the *C. striatum *isolates. Different clonal populations could be detected, as shown in Figure 1.

### Bacterial analysis by MALDI-TOF mass spectrometry

In the MALDI-TOF MS cluster analysis, the *Corynebacterium *species could be clearly differentiated from one another with less than 50% similarity. MALDI-TOF MS profiles for all of the strains studied have been included as Additional files 6: Figure S2. All the strains analysed clustered in four different groups (with similarities higher than 60%): the cluster of *C. striatum *included most of the clinical isolates and the type strain of *C. striatum*, and the cluster of *C. amycolatum *included the type strain, isolate CCUG 39137, the clinical isolate 70 (similarity higher than 60%), and two branches, including a single strain, the clinical isolate 69 and the environmental *Corynebacterium *CCUG 44705. The duplicate spectra for each strain analysed clustered at 60% similarity or higher. At a 70% similarity level, three subclusters could be distinguished in the *C. striatum *branch. Isolates 16 and 17 were identified as *C. pseudodiphtheriticum *by the RapID CB Plus^® ^strips, the method routinely used for identification in clinical laboratories, but they clustered within the *C. striatum *group in the MALDI-TOF analysis, in accordance with the sequencing analysis. These data further support that MALDI-TOF MS is an appropriate tool to differentiate and discriminate species, even at the level of expression of the most abundant cellular proteins.

## Discussion

Strains of *C. striatum *isolated from cultures of sputum of respiratory samples from patients with COPD were studied in order to find possible differences between them and the type strain. In general, this group of organisms is well identified by current phenotypic methods, but in some cases, there is a lack of specificity that may result in ambiguous or even erroneous identification. Correct identification of bacteria remains critical for the detection of outbreaks in specific populations of patients and for the surveillance of bacteria within patients.

Phenotypic characterisation and antibiotic-resistance profiles did not clearly distinguish between *C. striatum *strains. All strains were identifiable by the RapID CB Plus^® ^strips system, with three different identifications being generated. All identifications had confidence levels higher than 85.54%. Antibiotic-resistance profiles for *C. striatum *clinical isolates were heterogeneous in the different antibiotics tested, whereas the type strains of *C. amycolatum *and *C. striatum*, as well as the external controls analysed, were mainly susceptible to the antibiotics tested.

Differences within clinical *C. striatum *isolates were identified with PCR amplification and the sequencing of several genes. Of all the genes analysed, the ITS1 region and the *gyrA *and *rpoB *genes, due to their variability, were the most adequate to discriminate between strains, although ITS1 did not allow for calculations of genetic diversity because of the presence of more than one *rrn *operon. These genes were more polymorphic than the other genes tested. The analyses provided an appropriate identification of *C. striatum *strains and allowed for distinguishing between clinical isolates. Molecular analysis allows species discrimination, unlike phenotypic analysis, which sometimes misidentifies strains.

The 56 strains represent distinct allele combinations (19 STs, considering only three genes: ITS1, *gyrA *and *rpoB*); 11, 10, 6, and 6 strains showed identical allelic profiles (sequetypes 2, 4, 1 and 11, corresponding to the allelic profiles 6-2-2, 4-3-2, 3-2-2 and 7-3-3). All of the *C. striatum *clinical isolates were different from the type strain, and recombination events could be detected between them, supporting the hypothesis that these groups represent genetically similar strains.

The identification of strains based on molecular methods was also confirmed by MALDI-TOF mass spectrometry. The bacteria identified were exactly the same with both methods. As suggested by Seng *et al. *[15], MALDI-TOF may represent a rapid, inexpensive, alternative assay for identification of bacteria at the species level. These results were also in agreement with data obtained by Bittar *et al. *[8]. Our results suggest that MALDI-TOF mass spectrometry could also be a beneficial tool for discrimination of bacterial strains discrimination below the species level, but it is not as efficient as the molecular analysis for identifying strains. Further studies to evaluate the typing power should be performed.

## Conclusions

In summary, our results demonstrate that the isolates obtained were best identified with gene-based molecular methods and that they were different from the type strain of *C. striatum*. Additionally, the ITS1 region and the *gyrA *and *rpoB *genes are the most useful tools to discriminate between strains because of their variability, unlike the phenotype and antibiotype, which are not suitable for this purpose. Our results suggest that MALDI-TOF mass spectrometry is a good tool for *C. striatum *identification and for discriminating bacterial strains below the species level.

## Authors' contributions

MGo carried out the molecular genetic studies, participated in the sequence analysis and drafted the manuscript. FR coordinated samples collection and decided patient treatments. MCG and MGa carried out the isolation and phenotypic and the antibiogram analysis. FR, JBS and JL conceived the study. All co-authors participated in the design of the study and coordination and helped to the draft manuscript. All authors read and approved the final manuscript.

## Supplementary Material

Additional file 1 **Table S1**. List of isolates analysed, their origin and sample type. External strains for comparison purposes have been included in the study: the type strains *C. amycolatum *CCUG 35685^T ^and *C. striatum *ATCC 6940^T^, as well as two strains of *C. striatum *with different origins, CCUG 39137 (from a human wound) and CCUG 44705 (tobacco industry). Click here for file

Additional file 2 **Table S2**. Primers used for performing the molecular analysis of the 56 *Corynebacterium *strains. Click here for file

Additional file 3 **Table S3**. Phenotypic results of RapID CB Plus^® ^tests for the different strains analysed. Click here for file

Additional file 4 **Table S4**. Antibiotic susceptibility pattern of each strain analysed. The antibiotics tested for all strains were penicillin (PEN), imipenem (IMI), erythromycin (ERI), rifampicin (RIF), tetracycline (TET), vancomycin (VAN), ciprofloxacin (CIP), gentamicin (GEN), cefotaxime (CEF), and trimethoprim-sulfamethoxazole (TRI). R, resistant; I, intermediate; S, susceptible. Click here for file

Additional file 5 **Figure S1**. ERIC-PCR patterns of the different *C. striatum *clinical isolates analysed. The number on the top of the lane corresponds to the number of clinical isolate studied; Cs^T^, *C. striatum *ATCC 6940^T^. M1, Marker λ_E/H_; M2, marker 100 bp. Click here for file

Additional file 6 **Figure S2**. SARAMIS cluster analysis of all *Corynebacterium *strains isolated. Click here for file
