# Supplementary material for: Identification and diversity of multiresistant Corynebacterium striatum clinical isolates by MALDI-TOF mass spectrometry and by a multigene sequencing approach
Source: BMC Microbiol. 2012 Apr 4;12:52. doi: 10.1186/1471-2180-12-52 (PMC3348057; doi:10.1186/1471-2180-12-52)
Supplement: Additional file 1 — Table S1. List of isolates analysed, their origin and sample type. External strains for comparison purposes have been included in the study: the type strains C. amycolatum CCUG 35685T and C. striatum ATCC 6940T, as well as two strains of C. striatum with different origins, CCUG 39137 (from a human wound) and CCUG 44705 (tobacco industry). [file 1471-2180-12-52-S1.DOC]

Table S1. List of isolates analysed, their origin and sample type. External strains for comparison purposes have been included in the study: the type strains *C. amycolatum* CCUG 35685^T^ and *C. striatum* ATCC 6940^T^, as well as two strains of *C. striatum* with different origins, CCUG 39137 (from a human wound) and CCUG 44705 (tobacco industry).

| **Strain** | **Date** | **Sex** | **Hospital^a^** | **Sample** | **Quality of the sample^b^** | **Isolates per sample** |
| --- | --- | --- | --- | --- | --- | --- |
| 2 | May 2006 | Men | HSLL | Sputum | 1 | 1 |
| 7 | December 2006 | Men | HJM | Sputum | 1 | 2 (*C. striatum*, *Escherichia coli*) |
| 9 | January 2007 | Men | HJM | Sputum | 1 | 1 |
| 11 | February 2007 | Men | HJM | Sputum | 1 | 1 |
| 12 | April 2007 | Men | HJM | Sputum | 1 | 1 |
| 14 | February 2007 | Men | HJM | Sputum | 1 | 2 (*C. striatum*, *Pseudomonas aeruginosa*) |
| 15 | February 2007 | Men | HJM | Sputum | 1 | 1 |
| 16 | March 2007 | Men | HJM | Sputum | 1 | 1 |
| 17 | March 2007 | Men | HJM | Sputum | 1 | 1 |
| 18 | March 2007 | Men | HJM | Sputum | 2 | 2 (*C. striatum*, *P. aeruginosa*) |
| 19 | April 2007 | Men | HJM | Sputum | 1 | 2 (*C. striatum*, *Staphylococcus aureus*) |
| 21 | April 2006 | Men | HSLL | Sputum | 1 | 1 |
| 23 | March 2006 | Men | HJM | Sputum | 1 | 1 |
| 24 | March 2006 | Men | HJM | Sputum | 1 | 2 (*C. striatum*, *P. aeruginosa*) |
| 25 | June 2006 | Men | HJM | Sputum | 1 | 2 (*C. striatum*, *P. aeruginosa*) |
| 26 | March 2006 | Men | HJM | Sputum | 1 | 1 |
| 28 | May 2006 | Men | HSLL | Sputum | 1 | 2 (*C. striatum*, *Proteus mirabilis*) |
| 29 | November 2006 | Men | HJM | Sputum | 1 | 1 |
| 30 | November 2006 | Men | HJM | Sputum | 1 | 1 |
| 31 | December 2006 | Men | HJM | Sputum | 2 | 2 (*C. striatum*, *Stenotrophomonas maltophilia*) |
| 35 | February 2007 | Men | HJM | Sputum | 1 | 1 |
| 36 | February 2007 | Men | HJM | Sputum | 1 | 1 |

Table S1. Continued.

| **Strain** | **Date** | **Sex** | **Hospital^a^** | **Sample** | **Quality of the sample^b^** | **Isolates per sample** |
| --- | --- | --- | --- | --- | --- | --- |
| 41 | June 2007 | Men | HJM | Sputum | 1 | 1 |
| 42 | July 2007 | Men | HJM | Sputum | 1 | 2 (*C. striatum*, *P. aeruginosa*) |
| 43 | June 2007 | Men | HJM | Sputum | 1 | 1 |
| 44 | June 2007 | Men | HJM | Sputum | 1 | 1 |
| 46 | July 2007 | Men | HJM | Sputum | 1 | 1 |
| 47 | July 2007 | Men | HJM | Sputum | 2 | 3 (*C. striatum*, 2 *P. aeruginosa*) |
| 48 | March 2008 | Men | HJM | Sputum | 1 | 3 (*C. striatum*, *S. maltophilia*, *Moraxella catarrhalis*) |
| 50 | October 2007 | Men | HJM | Sputum | 1 | 1 |
| 51 | January 2008 | Men | HJM | Sputum | 1 | 2 (*C. striatum*, *P. aeruginosa*) |
| 53 | February 2008 | Men | HJM | Sputum | 1 | 3 (*C. striatum*, *P. aeruginosa*, *S. maltophilia*) |
| 54 | February 2008 | Women | HJM | Sputum | 1 | 2 (*C. striatum*, *P. aeruginosa*) |
| 55 | February 2008 | Men | HJM | Sputum | 1 | 2 (*C. striatum*, *P. aeruginosa*) |
| 56 | April 2008 | Men | HJM | Sputum | 1 | 2 (*C. striatum*, *P. aeruginosa*) |
| 57 | April 2008 | Men | HJM | Sputum | 1 | 1 |
| 58 | May 2008 | Women | HJM | Sputum | 1 | 1 |
| 59 | May 2008 | Men | HJM | Sputum | 2 | 3 (*C. striatum*, *P. aeruginosa*, *Achromobacter xylosoxidans*) |
| 60 | October 2008 | Women | HJM | Sputum | 2 | 1 |
| 61 | November 2008 | Women | HJM | Sputum | 1 | 1 |
| 62 | February 2009 | Women | HJM | Sputum | 1 | 1 |
| 63 | March 2009 | Women | HJM | Sputum | 1 | 2 (*C. striatum*, *P. aeruginosa*) |
| 64 | January 2009 | Men | HJM | Sputum | 1 | 1 |
| 65 | March 2009 | Men | HJM | Sputum | 2 | 2 (*C. striatum*, *Rothia mucilaginosa*) |
| 66 | February 2009 | Men | HG | Sputum | 1 | 3 (*C. striatum*, 2 *P. aeruginosa*) |
| 67 | February 2009 | Women | HJM | Sputum | 1 | 2 (*C. striatum*, *S. maltophilia*) |

Table S1. Continued.

| **Strain** | **Date** | **Sex** | **Hospital^a^** | **Sample** | **Quality of the sample^b^** | **Isolates per sample** |
| --- | --- | --- | --- | --- | --- | --- |
| 68 | February 2009 | Men | HJM | Dermis ulcer smear | 1 | 3 (*C. striatum/amycolatum*, *S. aureus*, *Enterobacter cloacae*) |
| 69 | March 2009 | Men | HSLL | Emergency - Sputum | 1 | 1 |
| 70 | March 2009 | Men | HSLL | Oncology – Surgical wound smear | 1 | 2 (*C. striatum/amycolatum*, *S. epidermidis*) |
| 71 | March 2009 | Men | HSLL | Emergency - Sputum | 2 | 1 |
| 73 | February 2009 | Men | HG | Sputum | 1 | 3 (*C. striatum*, 2 *P. aeruginosa*) |
| 74 | June 2009 | Men | HJM | Sputum | 1 | (*C. striatum*, *E. coli*) |

^a^ HSLL (Hospital Son LLàtzer), HJM (Hospital Joan March) and HG (Hospital General) are referred to different hospitals from Mallorca.

^b^Quality of the sample: 1, good quality; and 2, contamination with high number of leucocytes (>50).
