# Supplementary material for: Identification and diversity of multiresistant Corynebacterium striatum clinical isolates by MALDI-TOF mass spectrometry and by a multigene sequencing approach
Source: BMC Microbiol. 2012 Apr 4;12:52. doi: 10.1186/1471-2180-12-52 (PMC3348057; doi:10.1186/1471-2180-12-52)
Supplement: Additional file 2 — Table S2. Primers used for performing the molecular analysis of the 56 Corynebacterium strains. [file 1471-2180-12-52-S2.DOCX]

Table S2. Primers used for performing the molecular analysis of the 56 *Corynebacterium* strains.

| Gene | Primer | Sequence (5’🡪 3’) | Function | Tª (ºC) | Length (bp) | Reference |
| --- | --- | --- | --- | --- | --- | --- |
| 16S rDNA | 16SF27 | AGAGTTTGATCMTGGCTCAG | 16S rDNA | 55 | 1400 | [21] |
|  | 16SF357 | ACTCCTACGGGAGGCAGCAG |  |  |  |  |
|  | 16SR1492 | TACGGYTACCTTGTTACGACTT |  |  |  |  |
| ITS1 | 16F945 | GGGCCCGCACAAGCGGTGG | Interspacer sequence region 1 | 55 | 900 | [22] |
|  | 23r458 | CTTTCCCTCACGGTAC |  |  |  |  |
|  | rrn16S | GAAGTCGTAACAAGG |  |  |  |  |
| *gyrA* | gyrA1 | GCGGCTACGTAAAGTCC | Gyrase | 55 | 400 | [23] |
|  | gyrA2 | CCGCCGGAGCCGTTCAT |  |  |  |  |
| *rpoB* | C2700F | CGWATGAACATYGGBCAGGT | β-subunit of RNA polymerase enzyme | 60 | 400 | [24] |
|  | C3130R | TCCATYTCRCCRAARCGCTG |  |  |  |  |
| *hsp65* | TB11 | ACCAACGATGGTGTGTCCAT | Heat shock protein | 60 | 450 | [19] |
|  | TB12 | CTTGTCGAACCGCATACCCT |  |  |  |  |
| *sodA* | sodAF | ACGTTCACCACAGCAAGCACCA | Superoxid dismutase | 60 | 490 | [19] |
|  | sodAR | TCGGCCAGTTCACGACGTTCCA |  |  |  |  |
| *ermX* | ermXF | AACCATGATTGTGTTTCTGAACG | Erythromycin and clindamycin | 57 | 566 | [25] |
|  | ermXR | ACCAGGAAGCGGTGCCCT |  |  |  |  |
| *aphA* | aphAF | GGCAAGATCCTGGTATCGGTCT | Aminoglycoside | 57 | 480 | [25] |
|  | aphAR | AGACTAAACTGGCTGACGGCAT |  |  |  |  |
| *cmx* | cmxF | AGTCGGTATGGTCGTCGGC | Cloramphenicol  (2 identical subunits) | 57 | 879 | [25] |
|  | cmxR | GCTCCGATATTCAATGCTGCG |  |  |  |  |
| *repB* | repBF | CGATCTGGAATTTGTCTGCCGT | Replicase | 57 | 875 | [25] |
|  | repBR | CTGGTTGATAGACCCCGTGT |  |  |  |  |
| *tetA* | tetAF | TTAGCGTTCGGCGACCTGG | Tetracycline, oxytetracycline, and oxacillin | 58 | 1829 | [25] |
|  | tetAR | AACTGGGTGCCTTCAGGGTC |  |  |  |  |
