# Supplementary material for: Identification and diversity of multiresistant Corynebacterium striatum clinical isolates by MALDI-TOF mass spectrometry and by a multigene sequencing approach
Source: BMC Microbiol. 2012 Apr 4;12:52. doi: 10.1186/1471-2180-12-52 (PMC3348057; doi:10.1186/1471-2180-12-52)
Supplement: Additional file 3 — Table S3. Phenotypic results of RapID CB Plus® tests for the different strains analysed. [file 1471-2180-12-52-S3.DOC]

Table S3. Phenotypic results of RapID CB Plus® tests for the different strains analysed.

| **Strain** | **GLU** | **SUC** | **RIB** | **MAL** | **α-GLU** | **β-GLU** | **NAG** | **GLY1** | **ONPG** | **PHS** | **EST** | **PRO** | **TRY** | **PYR** | **LGLY** | **LEU** | **URE** | **NIT** | **CAT** | **PIG** | **Identification** |
| --- | --- | --- | --- | --- | --- | --- | --- | --- | --- | --- | --- | --- | --- | --- | --- | --- | --- | --- | --- | --- | --- |
| **2** | + | + | - | - | - | - | - | - | - | + | - | + | + | - | + | + | - | + | + | - | *C. striatum* (99.7 %) |
| **7** | + | + | - | - | - | - | - | - | - | + | - | + | + | - | + | + | - | + | + | - | *C. striatum* (99.7 %) |
| **9** | + | + | - | - | - | - | - | - | - | + | - | + | + | - | + | + | - | + | + | - | *C. striatum* (99.7 %) |
| **11** | + | + | + | - | - | - | - | - | - | + | + | + | + | - | + | + | - | + | + | - | *C. striatum* (99.62 %) |
| **12** | + | + | + | - | - | - | - | - | - | + | + | + | + | - | + | + | - | + | + | - | *C. striatum* (99.62 %) |
| **14** | + | + | - | - | - | - | - | - | - | + | + | + | + | - | + | + | - | + | + | - | *C. striatum* (99.99 %) |
| **15** | + | + | - | - | - | - | - | - | - | + | + | + | + | - | + | + | - | + | + | - | *C. striatum* (99.99 %) |
| **16** | - | - | - | - | - | - | - | - | - | - | - | + | + | - | + | + | + | + | + | - | *C. pseudodiphtheriticum* (100 %) |
| **17** | - | - | - | - | - | - | - | - | - | - | - | + | + | - | + | + | + | + | + | - | *C. pseudodiphtheriticum* (100 %) |
| **18** | + | + | - | - | - | - | - | - | - | + | + | + | + | - | + | + | - | + | + | - | *C. striatum* (99.99 %) |
| **19** | + | + | + | - | - | - | - | - | - | + | + | + | + | - | + | + | - | + | + | - | *C. striatum* (99.62 %) |
| **21** | + | + | - | - | - | - | - | - | - | - | - | + | + | - | + | + | - | + | + | - | *C. striatum* (99.97 %) |
| **23** | + | + | - | - | - | - | - | - | - | + | - | + | + | - | + | + | - | + | + | - | *C. striatum* (99.97 %) |
| **24** | + | + | - | - | - | - | - | - | - | + | - | + | + | - | + | + | - | + | + | - | *C. striatum* (99.97 %) |
| **25** | + | + | - | - | - | - | - | - | - | + | - | + | + | - | + | + | - | + | + | - | *C. striatum* (99.97 %) |
| **26** | + | + | - | - | - | - | - | - | - | + | - | + | + | - | + | + | - | + | + | - | *C. striatum* (99.97 %) |
| **28** | + | + | - | - | - | - | - | - | - | + | - | + | + | - | + | + | - | + | + | - | *C. striatum* (99.97 %) |
| **29** | + | + | - | - | - | - | - | - | - | + | - | + | + | - | + | + | - | + | + | - | *C. striatum* (99.97 %) |
| **30** | + | + | - | - | - | - | - | - | - | + | - | + | + | - | + | + | - | + | + | - | *C. striatum* (99.97 %) |
| **31** | + | + | - | - | - | - | - | - | - | + | - | + | + | - | + | + | - | + | + | - | *C. striatum* (99.97 %) |
| **35** | + | + | - | - | - | - | - | - | - | + | + | + | + | - | + | + | - | + | + | - | *C. striatum* (99.97 %) |
| **36** | + | + | - | - | - | - | - | - | - | + | + | + | + | - | + | + | - | + | + | - | *C. striatum* (99.99 %) |

Table S3. Continued.

| **Strain** | **GLU** | **SUC** | **RIB** | **MAL** | **α-GLU** | **β-GLU** | **NAG** | **GLY1** | **ONPG** | **PHS** | **EST** | **PRO** | **TRY** | **PYR** | **LGLY** | **LEU** | **URE** | **NIT** | **CAT** | **PIG** | **Identification** |
| --- | --- | --- | --- | --- | --- | --- | --- | --- | --- | --- | --- | --- | --- | --- | --- | --- | --- | --- | --- | --- | --- |
| **41** | + | + | - | - | - | - | - | - | - | + | + | + | + | - | + | + | - | + | + | - | *C. striatum* (99.99 %) |
| **42** | + | + | - | - | - | - | - | - | - | + | + | + | + | - | + | + | - | + | + | - | *C. striatum* (99.97 %) |
| **43** | + | + | - | - | - | - | - | - | - | + | - | + | + | - | + | + | - | + | + | - | *C. striatum* (99.97 %) |
| **44** | + | + | - | - | - | - | - | - | - | + | - | + | + | - | + | + | - | + | + | - | *C. striatum* (99.97 %) |
| **46** | + | + | - | - | - | - | - | - | - | + | - | + | + | - | + | + | - | + | + | - | *C. striatum* (99.97 %) |
| **47** | + | + | - | - | - | - | - | - | - | + | - | + | + | - | + | + | - | + | + | - | *C. striatum* (99.97 %) |
| **48** | + | + | - | - | - | - | - | - | - | + | - | + | + | - | + | + | - | + | + | - | *C. striatum* (99.97 %) |
| **50** | + | + | - | - | - | - | - | - | - | + | - | + | + | - | + | + | - | + | + | - | *C. striatum* (99.97 %) |
| **51** | + | + | - | - | - | - | - | - | - | + | + | + | + | - | + | + | - | - | + | - | *C. striatum* (85.54 %) |
| **53** | + | + | - | - | - | - | - | - | - | + | - | + | + | - | + | + | - | + | + | - | *C. striatum* (99.97 %) |
| **54** | + | + | - | - | - | - | - | - | - | + | - | + | + | - | + | + | - | + | + | - | *C. striatum* (99.97 %) |
| **55** | + | + | - | - | - | - | - | - | - | + | + | + | + | - | + | + | - | + | + | - | *C. striatum* (99.99 %) |
| **56** | + | + | - | - | - | - | - | - | - | + | + | - | + | - | + | + | - | + | + | - | *C. striatum* (99.99 %) |
| **57** | + | + | - | - | - | - | - | - | - | - | - | + | + | - | + | + | - | + | + | - | *C. striatum* (99.97 %) |
| **58** | + | + | - | - | - | - | - | - | - | - | - | + | + | - | + | + | - | + | + | - | *C. striatum* (99.97 %) |
| **59** | + | + | - | - | - | - | - | - | - | - | + | + | + | - | + | + | - | + | + | - | *C. striatum* (99.99 %) |
| **60** | + | + | - | - | - | - | - | - | - | + | - | + | + | - | + | + | - | + | + | - | *C. striatum* (99.97 %) |
| **61** | + | + | - | - | - | - | - | - | - | + | - | + | + | - | + | + | - | + | + | - | *C. striatum* (99.97 %) |
| **62** | + | + | - | - | - | - | - | - | - | + | - | + | + | - | + | + | - | + | + | - | *C. striatum* (99.97 %) |
| **63** | + | + | - | - | - | - | - | - | - | + | + | + | + | - | - | + | - | + | + | - | *C. striatum* (99.80 %) |
| **64** | + | + | - | - | - | - | - | - | - | - | - | + | + | - | + | + | - | + | + | - | *C. striatum* (99.95 %) |
| **65** | + | + | - | - | - | - | - | - | - | - | - | + | + | - | + | + | - | + | + | - | *C. striatum* (99.95 %) |
| **66** | + | + | - | - | - | - | - | - | - | - | - | + | + | - | + | + | - | + | + | - | *C. striatum* (99.95 %) |

Table S3. Continued.

| **Strain** | **GLU** | **SUC** | **RIB** | **MAL** | **α-GLU** | **β-GLU** | **NAG** | **GLY1** | **ONPG** | **PHS** | **EST** | **PRO** | **TRY** | **PYR** | **LGLY** | **LEU** | **URE** | **NIT** | **CAT** | **PIG** | **Identification** |
| --- | --- | --- | --- | --- | --- | --- | --- | --- | --- | --- | --- | --- | --- | --- | --- | --- | --- | --- | --- | --- | --- |
| **67** | + | + | - | - | - | - | - | - | - | - | - | + | + | - | + | + | - | + | + | - | *C. striatum* (99.95 %) |
| **68** | + | + | - | - | - | - | - | - | - | - | - | + | + | - | + | + | - | + | + | - | *C. striatum* (99.95 %) |
| **69** | + | + | - | + | - | - | - | - | - | - | - | + | + | - | + | + | - | + | + | - | *C. striatum* (99.99 %) |
| **70** | + | + | + | - | - | - | - | - | - | - | + | + | + | - | - | + | - | + | + | - | *C. striatum* (96.38 %) |
| **71** | + | + | - | - | - | - | - | - | - | - | - | + | + | - | + | + | - | + | + | - | *C. striatum* (99.95 %) |
| **73** | + | + | - | - | - | - | - | - | - | - | - | + | + | - | + | + | - | + | + | - | *C. striatum* (99.95 %) |
| **74** | + | + | - | - | - | - | - | - | - | + | + | + | + | - | + | + | - | + | + | - | *C. striatum* (99.90 %) |
| **ATCC 6940^T^** | + | + | - | - | - | - | - | - | - | - | - | + | + | - | + | + | - | + | + | - | *C. striatum* (99.95 %) |
| **CCUG 35685^T^** | + | - | - | - | - | - | - | - | - | - | - | + | + | - | - | - | + | + | + | - | *C. amycolatum* (51.26 %) |
| **CCUG 39137** | + | + | + | - | - | - | - | - | - | - | + | + | + | - | - | + | - | + | + | - | *C. striatum* (99.25 %) |
| **CCUG 44705** | + | + | + | - | - | - | - | - | - | - | - | + | + | - | + | + | - | + | + | - | *C. striatum* (97.09 %) |

¥ GLU, glucose; SUC, sucrose; RIB, ribose; MAL, maltose; α-GLU, p-nitrophenyl-α-D-glucoside; β-GLU, p-nitrophenyl- β-D-glucoside; NAG, p-nitrophenyl-N-acetyl-β-D-glucoside; GLY1, p-nitrophenyl glycoside; ONPG, o-nitrophenyl-β-D-galactopyranoside; PHS, phosphatase; EST, esterase; PRO, proline-β-naphthylamide; TRY, tryptophan-β-naphthylamide; PYR, pyrrolidine-β-naphthylamide; LGLY, leucyl-glycine-β-naphthylamide; LEU, leucine β-naphthylamide; URE, urease; NIT, nitrate reductase; CAT, catalase; PIG, pigment.
