# Supplementary material for: Identification and diversity of multiresistant Corynebacterium striatum clinical isolates by MALDI-TOF mass spectrometry and by a multigene sequencing approach
Source: BMC Microbiol. 2012 Apr 4;12:52. doi: 10.1186/1471-2180-12-52 (PMC3348057; doi:10.1186/1471-2180-12-52)
Supplement: Additional file 4 — Table S4. Antibiotic susceptibility pattern of each strain analysed. The antibiotics tested for all strains were penicillin (PEN), imipenem (IMI), erythromycin (ERI), rifampicin (RIF), tetracycline (TET), vancomycin (VAN), ciprofloxacin (CIP), gentamicin (GEN), cefotaxime (CEF), and trimethoprim-sulfamethoxazole (TRI). R, resistant; I, intermediate; S, susceptible. [file 1471-2180-12-52-S4.DOC]

Table S4. Antibiotic susceptibility pattern of each strain analysed. The antibiotics tested for all strains were penicillin (PEN), imipenem (IMI), erythromycin (ERI), rifampicin (RIF), tetracycline (TET), vancomycin (VAN), ciprofloxacin (CIP), gentamicin (GEN), cefotaxime (CEF), and trimethoprim-sulfamethoxazole (TRI). R, resistant; I, intermediate; S, susceptible.

| **Strain** | **PEN** | **IMI** | **ERI** | **RIF** | **TET** | **VAN** | **CIP** | **GEN** | **CEF** | **TRI** |
| --- | --- | --- | --- | --- | --- | --- | --- | --- | --- | --- |
| **2** | I | I | R | R | S | S | R | S | R | R |
| **7** | I | R | S | R | S | S | R | S | R | R |
| **9** | I | R | I | R | R | S | R | I | R | R |
| **11** | S | S | R | R | S | S | R | R | R | R |
| **12** | S | S | R | R | S | S | R | R | R | R |
| **14** | S | R | R | R | R | S | R | I | R | R |
| **15** | I | R | R | R | R | S | R | I | R | R |
| **16** | I | I | R | R | R | S | R | I | R | R |
| **17** | S | S | R | R | S | S | R | I | R | R |
| **18** | R | R | R | R | R | S | R | R | R | R |
| **19** | R | R | R | R | S | S | R | R | R | R |
| **21** | R | R | S | R | S | S | R | S | R | R |
| **23** | I | R | S | R | R | S | R | S | R | R |
| **24** | I | R | S | R | S | S | R | R | R | R |
| **25** | I | R | R | R | S | S | R | S | R | R |
| **26** | I | R | R | R | R | S | R | I | R | R |
| **28** | R | R | R | R | S | S | R | I | R | R |
| **29** | I | S | R | R | S | S | R | S | R | R |
| **30** | R | R | R | S | S | S | R | S | R | R |
| **31** | I | R | S | R | R | S | R | I | R | R |
| **35** | I | R | R | R | R | S | R | S | R | R |
| **36** | I | R | R | R | R | S | R | S | R | R |
| **41** | I | R | S | R | R | S | R | S | R | R |
| **42** | I | R | S | R | R | S | R | S | R | R |
| **43** | S | S | R | R | R | S | R | S | R | R |
| **44** | R | R | R | R | R | S | R | I | R | R |
| **46** | I | R | R | R | R | S | R | I | R | R |
| **47** | R | R | R | R | R | S | R | I | R | R |
| **48** | S | S | R | R | S | S | R | I | R | R |
| **50** | I | R | I | R | R | S | R | S | R | R |
| **51** | R | S | R | R | I | S | R | S | R | R |
| **53** | I | S | R | R | S | S | R | S | R | R |
| **54** | S | S | S | R | S | S | R | S | R | R |
| **55** | R | R | I | R | R | S | R | S | R | R |
| **56** | R | R | R | R | S | S | R | R | R | R |
| **57** | R | R | R | R | I | S | R | S | R | R |
| **58** | R | R | R | R | I | S | R | S | R | R |
| **59** | R | S | R | R | I | S | R | S | R | S |
| **60** | S | S | R | R | R | S | R | S | R | R |
| **61** | I | S | R | S | R | S | R | I | R | R |

Table S4. Continued.

| **Strain** | **PEN** | **IMI** | **ERI** | **RIF** | **TET** | **VAN** | **CIP** | **GEN** | **CEF** | **TRI** |
| --- | --- | --- | --- | --- | --- | --- | --- | --- | --- | --- |
| **62** | S | S | R | S | R | S | R | S | R | R |
| **63** | S | S | R | S | R | S | R | S | R | R |
| **64** | R | S | R | S | S | S | R | S | R | S |
| **65** | I | S | R | S | S | S | R | S | R | I |
| **66** | R | S | R | S | S | S | R | S | R | R |
| **67** | S | S | R | S | R | S | R | S | R | R |
| **68** | S | S | R | S | S | S | R | S | R | S |
| **69** | S | S | S | S | S | S | R | S | R | S |
| **70** | R | R | S | S | S | S | R | S | R | R |
| **71** | R | R | R | R | S | S | R | S | R | R |
| **73** | R | S | R | S | S | S | R | S | R | R |
| **74** | R | R | R | S | S | S | R | S | R | S |
| **ATCC 6940^T^** | S | S | S | S | S | S | S | S | R | S |
| **CCUG 35685^T^** | S | S | S | S | S | S | S | S | S | S |
| **CCUG 39137** | I | S | S | S | S | S | R | S | S | S |
| **CCUG 44705** | S | S | I | S | S | S | S | S | S | S |
