# Supplementary material for: Identification and diversity of multiresistant Corynebacterium striatum clinical isolates by MALDI-TOF mass spectrometry and by a multigene sequencing approach
Source: BMC Microbiol. 2012 Apr 4;12:52. doi: 10.1186/1471-2180-12-52 (PMC3348057; doi:10.1186/1471-2180-12-52)
Supplement: Additional file 5 — Figure S1. ERIC-PCR patterns of the different C. striatum clinical isolates analysed. The number on the top of the lane corresponds to the number of clinical isolate studied; CsT, C. striatum ATCC 6940T. M1, Marker λE/H; M2, marker 100 bp. [file 1471-2180-12-52-S5.DOC]

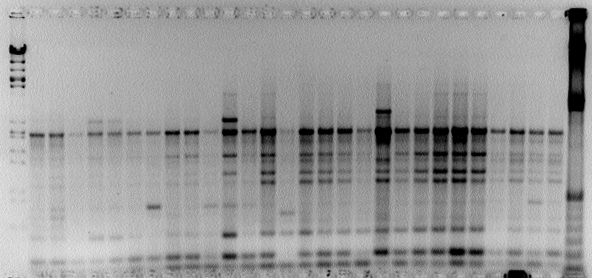


M1 2 7 9 11 12 14 15 16 17 18 19 21 23 24 25 26 28 29 31 35 36 41 42 43 44 45 46 47 M2

M1 48 50 51 53 54 55 56 57 58 59 60 61 62 63 64 65 66 67 68 69 70 71 72 73 Cs^T^M2


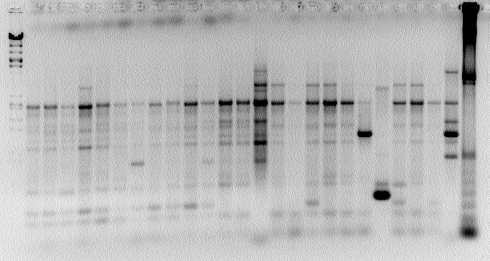


Figure S1. ERIC-PCR patterns of the different *C. striatum* clinical isolates analysed. The number on the top of the lane corresponds to the number of clinical isolate studied; Cs^T^, *C. striatum* ATCC 6940^T^. M1, Marker λ_E/H_; M2, marker 100 bp.
