# Supplementary figures and images for: Identification and diversity of multiresistant Corynebacterium striatum clinical isolates by MALDI-TOF mass spectrometry and by a multigene sequencing approach
Source: BMC Microbiol. 2012 Apr 4;12:52. doi: 10.1186/1471-2180-12-52 (PMC3348057; doi:10.1186/1471-2180-12-52)

Figure S2. SARAMIS cluster analysis of all *Corynebacterium* strains isolated.


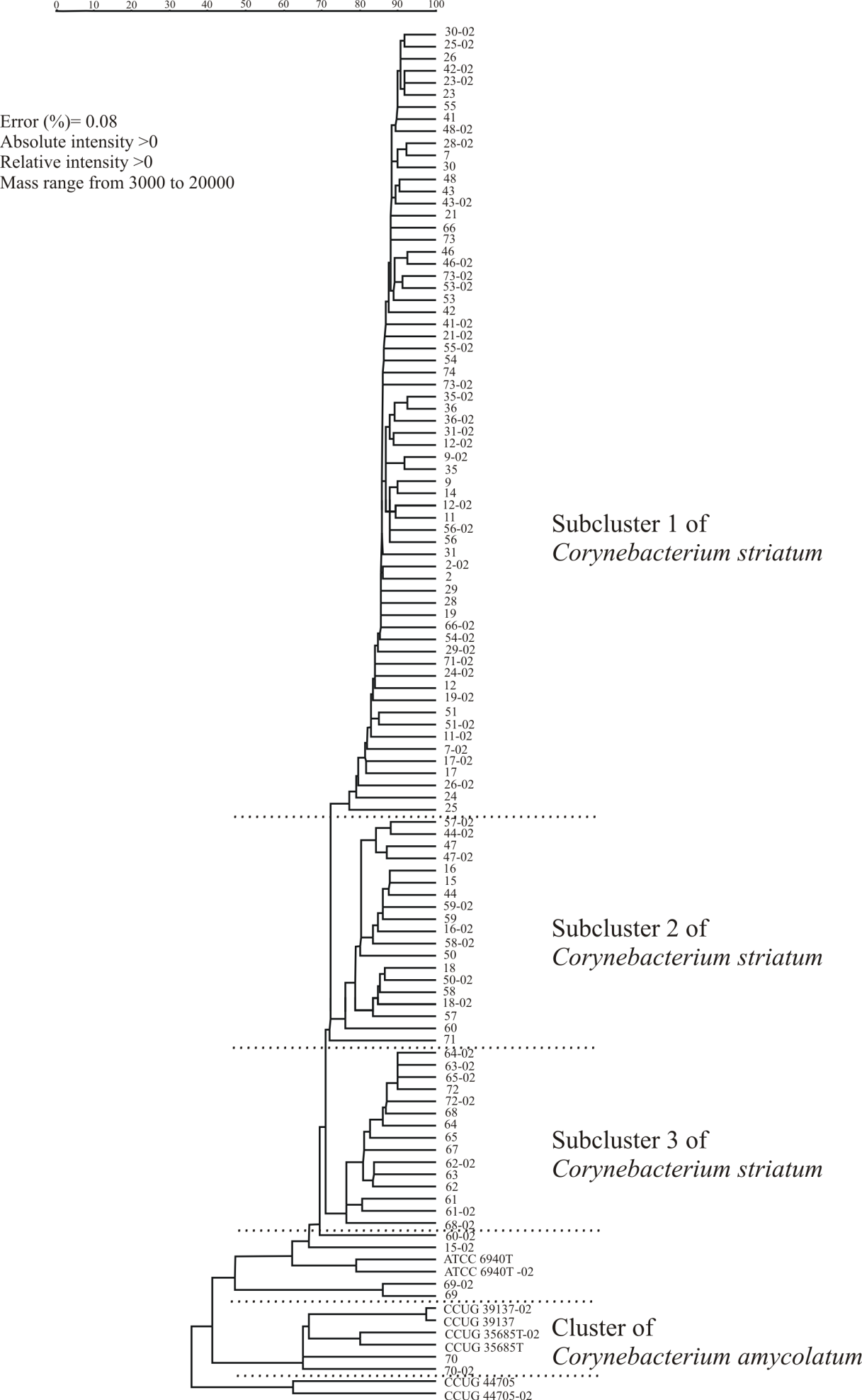

Supplement: Additional file 6 — Figure S2. SARAMIS cluster analysis of all Corynebacterium strains isolated. [file 1471-2180-12-52-S6.DOC]
